# The uses of Kenyan aloes: an analysis of implications for names, distribution and conservation

**DOI:** 10.1186/s13002-015-0060-0

**Published:** 2015-11-25

**Authors:** Charlotte S. Bjorå, Emily Wabuyele, Olwen M. Grace, Inger Nordal, Leonard E. Newton

**Affiliations:** Natural History Museum, University of Oslo, P.O. Box 1172, Blindern, 0318 Oslo Norway; Department of Bioscience, University of Oslo, P.O. Box 1066, Blindern, 0316 Oslo Norway; Department of Plant Sciences, Kenyatta University, P.O. Box 43844, Nairobi, 00100 Kenya; Comparative Plant & Fungal Biology, Royal Botanic Gardens, Kew, Surrey TW9 3AB UK

**Keywords:** *Aloe*, Ethnobotany, Ethnotaxonomy, Folk use, Conservation, Kenya, Plant use

## Abstract

**Background:**

The genus *Aloe* is renowned for its medicinal and cosmetic properties and long history of use. Sixty-three *Aloe* species occur in Kenya, of which around 50 % are endemic. Several species of aloes are threatened with extinction and knowledge about their use is of major importance for sound conservation strategies. The main aims of this study were to assess the biocultural value of *Aloe* in Kenya by documenting local uses of aloes and evaluating how the vernacular names reflect the relative importance in different ethnic groups.

**Methods:**

Ethnobotanical and ethnotaxonomical data were collected using field observations and semi-structured interviews. Information was collected by interviewing 63 respondents from nine different ethnic groups, representing different ages, gender and occupations. Statistical analyses were performed using R version 3.1.2.

**Results:**

A total of 19 species of *Aloe* were found in the study area, of which 16 were used. On the generic level *Aloe* was easily distinguished. At species level, the local and scientific delimitation were almost identical for frequently used taxa. *Aloe secundiflora*, with 57 unique use records was the most important species. The two most frequently mentioned *Aloe* treatments, were malaria and poultry diseases. In our study area neither age nor gender had a significant influence on the level of knowledge of *Aloe* use. Finally, no correlation was found between extent of use and people’s perception of decrease in local aloe populations. The aloes are highly appreciated and are therefore propagated and transported over large areas when people relocate.

**Conclusion:**

Biocultural value is reflected in the ethnotaxonomy of *Aloe* in Kenya. Different ethnic groups recognise their most-valued *Aloe* at the genus level as “the aloe” and add explanatory names for the other species, such as the “spotted aloe” and the “one-legged aloe”. Widespread species of *Aloe* have the highest number of uses. There is no obvious correlation with high use and decrease in abundance of aloes locally, and we found no compelling evidence for local uses causing devastating damage to populations of the 19 species in use, whereas habitat loss and commercial harvesting appear to be of urgent concern for these important plants.

## Background

The genus *Aloe* L. is renowned for its medicinal and cosmetic properties that have been exploited over millennia [1–4], including the popular species *Aloe vera* (L.) Burm.f. which was recorded in use as early as 400 BCE [5]. The genus contains around 550 species [6] but only a few species feature in international trade of any significance [7], i.e. *Aloe vera*, *A. perryi* Baker, *A. ferox* Mill. and *A. arborecens* Mill.

Kenya is known for its high diversity of aloes [8–11]. Carter et al. [8] reported 59 species in Kenya, and since then four more species have been described [12, 13]. Due to their multi-use nature, Kenyan aloes have in been harvested at household level over many years mainly for use as herbal remedies in combination with other plants [14]. The commercial exploitation of aloes in Kenya was first reported in the 1960s, when substantial amounts of wild-harvested leaf extracts, mainly of *A. secundiflora* Engl., were exported to the USA [15]. Since then, several similar incidents of wild harvested aloe extracts for sale have been reported [16, 17]. In 1986, the then president of Kenya declared a ban on harvesting wild-growing aloes in recognition of the potentially devastating effects of harvesting on the plants and their habitats [18]. However, the presidential decree was not translated into a legal instrument and was largely ignored [16]. In some areas where the law was adhered to, it led to more harm than protection [19]: rather than defoliating plants in natural populations and allowing recovery, plants of *Aloe secundiflora* were dug up and re-planted in “plantations”. In 2007 stakeholders consulted widely and came up with Aloe Utilization guidelines that were gazetted by the Kenya government at the end of 2007 as subsidiary legislation [20], bridging the legal gap in the aloe industry.

In Kenya today, the species *A. secundiflora, A. turkanensis* Christian*, A. rivae* Baker*, A. calidophila* Reynolds, and *A. scabrifolia* L.E.Newton & Lavranos are reported to be commercially exploited for aloe bitter exudate [17, 21]. *A. scabrifolia* and *A. turkanensis* [16] are particularly threatened by organized collecting activities coordinated by dealers, who have trained local communities in *Aloe* processing techniques, to supply illegal export of large quantities of dried exudate [21, 22]. This trade is sustained by the global demand for *Aloe* products [7]. Another, less specific threat to the aloes is the general loss and destruction of habitats due to increase in human and livestock populations (e.g. [23]). In addition to national protection in Kenya, aloes and their products are regulated in international trade by the Convention on International Trade in Endangered Species (CITES) and are all listed on CITES appendix II [9]. The IUCN Red List [24], furthermore, records only seven species of *Aloe* in three most threatened categories ‘Critically endangered’, ‘Endangered’ or “Vulnerable”. The 25 endemic *Aloe* species of Kenya were evaluated by Wabuyele et al. [11], who concluded that no fewer than 16 species were either ‘Critically endangered’ or ‘Endangered’, according to IUCN Red List criteria. In spite of growing awareness for the threats to *Aloe* species in Kenya and these attempts to protect them, illegal harvesting is still taking place. Although there is evidence of an ongoing demand for the processed aloe exudate, the (legal) non-commercial uses of *Aloe* species in rural communities, and the sustainability of local *Aloe* harvesting, have not been recently assessed.

Kenya’s drylands support 28 % of the total human population and occupy 80 % of the land area. Sustainable utilization of *Aloe* resources in the drylands has been advocated [17] as a step towards empowering the local communities for better livelihoods and, indirectly, biodiversity conservation. Knowledge about the uses of aloes is crucial to help prioritise conservation and research efforts towards the most valued and threatened species. Plant value, in particular, can be a convincing argument for conservation attention, but is difficult to quantify since folk uses and cultural significance are rarely evaluated in economic terms [7]. Grace et al. [4] argued that a variety of indicators can be used to estimate the cultural and economic value of *Aloe,* such as the number of vernacular names and number of uses recorded for a species. Holman [25] demonstrated that the ways local communities name plants reveals important information about the plants’ relative importance to the community. Earlier studies have shown that in South Africa most species of *Aloe* are used [26], and the same has been reported about aloes in Kenya [16]. In this study, we aimed to assess the contemporary biocultural importance of *Aloe* species in Kenya, using a case study to examine the uses, vernacular names and sustainability of non-commercial wild harvesting and gather perceptions of population changes among nine ethnic groups in Kenya. Specifically, the aims of this study were to document the vernacular names and local uses of aloes in Kenya, explore the distribution of local knowledge about *Aloe* species in relation to age and gender, and record people’s understanding of changes in abundance and distribution of aloes.

## Methods

### Study area

**Fig. 1:**
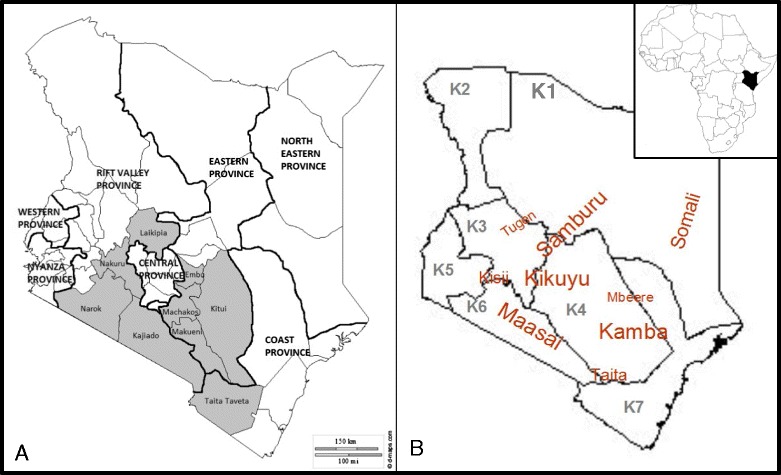
**a.** Map of Kenya showing study area (highlighted). **b.** Flora regions following *Flora of Tropical East Africa* (FTEA) and ethnic groups represented in the study area. Label size indicates the size of the ethnic group

The study area (Fig. 1a) was selected to areas in Kenya with a minimum of two *Aloe* taxa present, and areas of very low human population density, like Northern Kenya, were avoided. The study area was further selected to include a wide range of ethnic groups, representing different linguistic groups: Cushitic, Nilotic and Bantu (Fig. 1b). When this fieldwork was undertaken, Kenya comprised eight provinces (Swahilia: *mikoa*), that were subdivided into 69 districts (*wilaya*) and 497 divisions (*taarafa*). Under the new constitution (envisioned by the 2010 Constitution of Kenya), the former subdivisions were abandoned, and Kenya is now divided into 47 counties. Counties where information was collected are highlighted in grey (Fig. 1a), with the old provinces indicated.

**Fig. 2:**
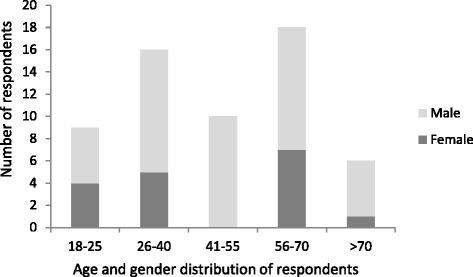
Distribution of the respondent’s age and gender

The ethnobotanical survey was undertaken during August and September 2005. Information was collected by interviewing 63 respondents from nine ethnic groups (Kamba, Kikuyu, Kisii, Masaii, Mbeere, Taita, Tugen, Samburu, Somali). Informants were selected to represent a broad spectrum of the communities, ages, gender and occupations, from herders to herbalists. The two youngest informants were 18 years old, the oldest estimated her age to around 100 years; the age and gender of informants are presented in Fig. 2. Semi-structured interviews were conducted with each respondent while walking in *Aloe* localities in the vicinity of the respondents’ homes. The information collected included vernacular names of plants, plant uses, harvesting procedures and preparation methods, plant population abundance, and whether changes had been registered through time. Where abundance was said to have changed, the informants were asked to suggest reasons for this. To get an indication of the informants’ general knowledge of the area, they were asked about issues such as land ownership, uses of other plants and ecological information like flowering times. Finally, the respondents were asked to rank the locally occurring *Aloe* species according to the single criterion of importance to themselves, reflecting the overall value they placed on different *Aloe* species.

To organise the information, the unique use records were categorised into the following classes: medicinal (humans), veterinary, fodder, local brew, fencing and soil stabilising, cosmetic, and “other” uses. All unique use records are listed in Appendix 1. Only specific uses were recorded, if respondents reported e.g. “this aloe is used to treat animals against diseases”, this was not counted, as it was too vague to distinguish it from other uses. Vouchers for all species are deposited in East African (EA) and or Oslo herbaria (O).

Information on the distribution and uses of aloes was collated from existing literature e.g. [6, 8, 10, 12, 13, 27–32], as well as specimens deposited at the East African Herbarium (EA) and the Herbarium at the Royal Botanic Gardens, Kew (K).

### Statistical analyses

Statistical analyses of the data were performed using R version 3.1.2.for Windows [33]. To test if there was any correlation between the age of the informant and their knowledge of use of aloes (unique use records), the non-parametric Kendall’s τ test was performed. To test if there was a significant difference between the informant’s gender and knowledge of use of aloes the non-parametric Wilcoxon-Mann–Whitney *U*-test was performed. These tests were both based on 58 observations. To test if the number of unique use records was correlated with the distribution of the aloe species, a Kendall’s τ test was performed based on 19 observations. To see if there was a connection between number of unique use records and people’s perception of decrease in local aloe populations we did a Wilcoxon-Mann–Whitney *U*-test based on 73 observations.

## Results

### Names - ethnotaxonomy

A total of 19 *Aloe* species were encountered in the study area. Vernacular names are presented in Table 1 with reference to the scientific names. All nine ethnic groups in this study had a name more or less equivalent to the scientific genus name, e.g. “suguroi” (Samburu), osuguroi (Maasai), cheretwo (Tugen), kiluma (Kamba) (Table 1). These names were constant within the ethnic groups over extensive geographical areas. Little or no variation in the names was observed at the generic level, whereas at lower taxonomic levels we observed more variation and less stability in names. Sometimes different names were used for two populations of the same species; this was often linked to morphological variation. For example, if the plants in one population of *Aloe secundiflora* had spotted leaves in one locality, but not in another, these were often named differently, as kiluma (*A. secundiflora*, not spotted) and kiluma kila kimaa (*A. secundiflora*, spotted) in the Kamba language. The first, with no additional name, was often referred to as the “real” kiluma.

**Table 1:** Ethnotaxonomical naming of *Aloe* species by nine ethnic groups in Kenya

Scientific name	Language: (linguistic classification)	Local name(s)	Translation
***A. aageodonta***	Kamba (B)	kiluma	aloe
*A. ballyi*	Mberee (B)	kithunju	aloe
	Taita (B)	kipapa	aloe
***A. classenii***	Taita (B)	kipapa	aloe
***A. chrysostachys***	Mberee (B)	kithunju	aloe
***A. deserti***	Kamba (B)	kiluma	
	Maasai (N)	(o)suguroi lengejonabo	The one legged aloe
*A. elata*	Maasai (N)	(o)suguroi lengejonabo	The one legged aloe
***A. fibrosa***	Kamba (B)	kiluma	aloe
***A. kedongensis***	Maasai (N)	(o)suguroi	aloe
		(o)suguroi onyokie	The greyish aloe
		(o)suguroi olongapeta	The aloe of long post
	Kikuyu (B)	munywanugu	The aloe eaten by baboons
	Kisii (B)	omugaka	aloe
	Tugen-Aror (N)	cheretwo	aloe
*A. lateritia*	Kamba (B)	Kiluma	aloe
		Kiluma kila kimaa	The spotted aloe
	Kikuyu (B)	Kil(/r)uma	aloe
	Kisii (B)	Omugaka	aloe
	Maasai (N)	(o)suguroi	aloe
	Samburu (N)	suguroi lekoshe	aloe
		suguroi mara	The spotted aloe
		suguroi sambu	The spotted aloe
	Somali (C)	warabe	aloe
	Taita (B)	kipapa	aloe
	Tugen (N)	tangaratweti	aloe
	Tugen-Aror (N)	cheretwo	aloe
*A. morijensis*	Maasai (N)	(o)suguroi	aloe
		(o)suguroi lekpo	The aloe of down
***A. murina***	Maasai (N)	(o)suguroi	aloe
*A. ngongensis*	Samburu (N)	suguroi	aloe
		suguroi lengejonabo	The one legged aloe
*A. ngongensis* (cont.)		suguroi keri	The purple/ greyish aloe
	Taita (B)	kipapa	aloe
***A. nyeriensis***	Samburu (N)	suguroi lodo/yodo	The tall aloe
		suguroi mara	The spotted aloe
		suguroi ngare	The aloe of goats
***A. penduliflora***	Taita (B)	kipapa	aloe
***A. scabrifolia***	Samburu (N)	suguroi	aloe
*A. secundiflora*	Kikuyu (B)	Kil(/r)uma	aloe
	Maasai (N)	(o)suguroi	aloe
		(o)suguroi kianwan	The real aloe
		(o)suguroi kirimo	The spotted aloe
		(o)suguroi lengiok	The large leaved aloe
		(o)suguroi lenesho/lenaisho	aloe of beer
		(o)suguroi lenkejunabo	The one legged aloe
		(o)suguroi onyori	The green aloe
		(o)suguroi orok	The short aloe
		lnkoisikirianchoi	aloe
	Mbeere (B)	githunju/kithunju	aloe
	Samburu (N)	suguroi	aloe
		suguroi mara	The spotted aloe
		suguroi orok/yorok	The black aloe
	Somali (C)	dahr	aloe
	Taita (B)	kipapa	aloe
	Tugen-Aror (N)	cheretwo	aloe
***A. ukambensis***	Kamba (B)	kiluma	aloe
		kiluma kya ivia	The aloe of the rock
***A. vituensis***	Samburu (N)	no name	
*A. volkensii*	Maasai (N)	(o)suguroi	aloe
		(o)suguroi lombokishi	The aloe of long post

Species in bold are endemic to Kenya. Linguistic classifications *C* Cushitic, *N* Nilotic and *B* Bantu

When more than one *Aloe* taxon was identified within an area, a name in addition to the generic name was added (rather like the binomial scientific nomenclature) to describe the taxon it referred to. For instance, among the Samburu, if plants of *A. secundiflora*, which is stemless or has a very short stem, grows in the vicinity of the tall *A. ngongensis* Christian, *A. ngongensis* would be referred to as suguroi lengejonabo (one legged aloe), while *A. secundiflora* would be referred to as suguroi. In other examples, the naming was also linked to use, such as osuguroi lenesho (Maasai), meaning the aloe of beer. Sometimes the same name was used for different scientifically recognized taxa, e.g. *suguroi mara*, (the spotted aloe) was the name for a spotted *A. secundiflora* in one area, while it was referred to a heavily spotted *A. laterita* Engl. in another area.

### Local uses - ethnobotany

**Fig. 3:**
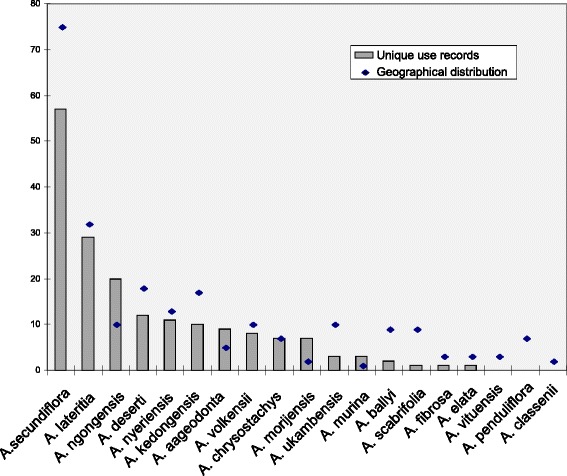
Number of unique uses for each *Aloe* species in the study area and geographical range represented by the number of recorded localities for the species from the East African-, Kew- and Oslo-herbaria

**Fig. 4:**
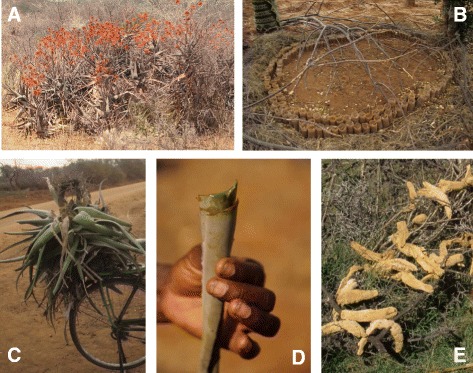
**a** *Aloe kedongensis* bearing the local name “suguroi onyokie” –the grey aloe*.* **b** Local propagation of *Aloe secundiflora*: seedlings are fenced to avoid browsing. **c** Collection of wild *Aloe secundiflora* for cultivation. **d** *Aloe secundiflora* is the most commonly used *Aloe* in Kenya. **e** *Aloe secundiflora* roots stripped and dried to be used in local beer production, and thus named “suguroi lenesho” – aloe of beer

Among the 19 species of *Aloe* found in the study area, the highest number of unique uses were recorded for *A. secundiflora* (Figs. 3, 4d). All respondents, irrespective of ethnic group, ranked this species as the most important. The informants mentioned twice as many unique uses for this species compared to the second most-used species, *A. lateritia.*

Altogether, 57 unique uses were mentioned for *Aloe secundiflora,* of which about 70 % were for medicinal uses in humans and livestock (Appendix 1). This species was also the most popular in most categories of use, except for cosmetic use, where *A. lateritia* was more frequently cited. Aloes were often mentioned as fodder plants, and *Aloe ngongensis* was as important as *A. secundiflora* for this purpose. The plants are browsed during drought, when other fodder plants are scarce; this was the case in many localities, over several ethnic groups. The importance of *Aloe nyeriensis* as a fodder plant among the Samburu and Masai is demonstrated by its vernacular name: aloe of goats (suguroi ngare).

Malaria was the most frequently mentioned ailment that aloes were used to treat. People did not use it uncritically however; being aware it might be lethal taken under certain circumstances (Appendix 1). In the current study 50 % (nine species) were used for this purpose by eight different ethnic groups. The second most mentioned use was for treatments of poultry diseases; seven different ethnic groups used six species for this purpose (Appendix 1).

Besides the similarities in uses recorded for each species of *Aloe*, some uses were linked to only one or a few ethnic groups, and were not widely shared across the ethnic groups surveyed. One of the most frequent uses, mentioned by informants from the Samburu and Masai ethnic groups, was the use of *Aloe secundiflora* for making traditional beer. The roots are dug up, stripped, soaked in honey and dried before being processed further for improving fermentation (Fig. 4e).

For 40 of the *Aloe* species in Kenya we did not find any documentation of use; as many as 22 of these are endemic species only found within a restricted area. Species such as *Aloe amicorum* L.E.Newton and *A. kulalensis* L.E.Newton & Beentje are found in remote areas almost inaccessible to people or at great distances from where people live. Their geographical isolation is a likely reason for the lack of documented uses.

### Distribution of knowledge

In this study, no significance in the level of knowledge between older and younger people was found (τ = 0.0727, P = 0.4380), nor were there any significant differences between the genders (W = 364.5, P = 0.7906). The Maasai and Kamba ethnic groups listed highest numbers unique use records for the *Aloe* species (Appendix 1).

### Popular perception of change in aloe abundance

Older respondents were more likely to report perceptible decreases in *Aloe* availability and abundance than younger respondents. However, no correlation was found between species with a high number of unique use records and those with a perceived decline in population size (W = 587, P = 0.6230). On the contrary, in some cases, high levels of use tended to increase the local abundance and availability of a species since people actively cultivated the plants (Fig. 4). In the Taita Hills, one of the informants had a nursery for *Aloe secundiflora* for use in the commercial production of soap (Fig. 4b). When collecting ethnobotanical data during this study, several historic *Aloe* localities (including type localities) were visited. However many species were not found, often due to habitat destruction and the loss of the locality, supporting mounting concerns [11] for the conservation of *Aloe* in Kenya.

## Discussion

Aloes are easily distinguished from other plants by their characteristic succulent spiny leaves, and thus it is not surprising that the local naming at the genus level is consistent with the scientific species concepts. However, a species may be given two names to reflect the appearance or use of the plants. When two species have the same vernacular name, they also share the way of use, and the local people therefore have no practical need to distinguish the species.

The vernacular names of *Aloe* species in Kenya are closely linked to use. In all areas where the widespread and widely used *A. secundiflora* occurred, this species was referred to as “the aloe” or “the real aloe”. In our study region, relatively few species of *Aloe* co-occur, making the recognition of *Aloe secundiflora* sufficient to distinguish the other species from “the real one” by descriptive characteristics like “the tall aloe” or “the grey aloe”. This naming also reflects the importance of the different species to the local communities; the “real aloe” is the most important aloe, and the other species are less important. If an aloe is not of any specific use, it is often not given any name at all. When the respondents were asked for names of some these species, they responded: “it is an aloe, but not a real one”. Our observations of vernacular nomenclature applied to *Aloe* spp. and overlap with scientific species concepts agree broadly with previous studies that have reported a binomial system in which vernacular names describe the plant name and its use [34, 35].

*Aloe secundiflora* and *A. lateritia* were the most important *Aloe* species in our study area according to the respondents, and this is evident in the wide variety of uses listed for these species. Several informants said, when describing the uses of *Aloe lateritia*, that it was used when *A. secundiflora* was not available. They further claimed that the *Aloe lateritia* is not as bitter as *A. secundiflora*, and hence not as effective. These two species are the most widespread in our study area, and thus have the greater potential of being used compared to other more geographically restricted species (Fig. 3). The different traditions of the nine ethnic groups surveyed contributed to increasing the diversity of uses recorded for these widespread species. *Aloe secundiflora* and *A. lateritia* are the only species of *Aloe* that are found in almost all of the seven Flora regions (*Flora of Tropical East Africa K1*- K7 Fig. 1b, [8])*.* Most aloes in Kenya are geographically restricted, but might at the same time be locally common [8, 11]. This is the case for species like *Aloe aageodonta* L.E.Newton and *A. chrysostachys* Lavranos & L.E.Newton, which are found only in the Flora region K4. The respondents used these species in more or less the same way as *Aloe secundiflora* in other areas. But where the two species (*Aloe secundiflora* and *A. lateritia*) were readily available, these were mostly preferred. Interestingly, other ethnobotanical studies elsewhere in Kenya and Uganda reported that the use of mixtures of medicinal plants, including *Aloe* spp., is much less common than the use of single-species preparations [35–37]. Poisonous members of the genus, like *Aloe ballyi* Reynolds and also *A. elata* S.Carter & L.E.Newton, which are used by people for arrow poison, are known to contain toxic hemlock alkaloid γ-coniceine [29], are generally avoided by humans and cattle.

Two uses were emphasized by respondents in each of the nine ethnic groups: the use of *Aloe* species for the treatment of malaria and for poultry diseases. These findings are supported by pharmacological studies e.g. [30, 31] and ethnobotanical studies in Kenya that have reported malaria as the greatest medical concern treated with herbal remedies e.g. [35]. Similarly, aloes feature prominently in ethnoveterinary medicine throughout their range and are most valued for their uses against insect pests [26, 34, 36]. The relative importance of Aloe in ethnomedicine and ethnoveterinary medicine should be understood in the regional context; in some areas of East Africa, herbal treatments are the only option for up to 80 % of the population [14].

**Fig. 5:**
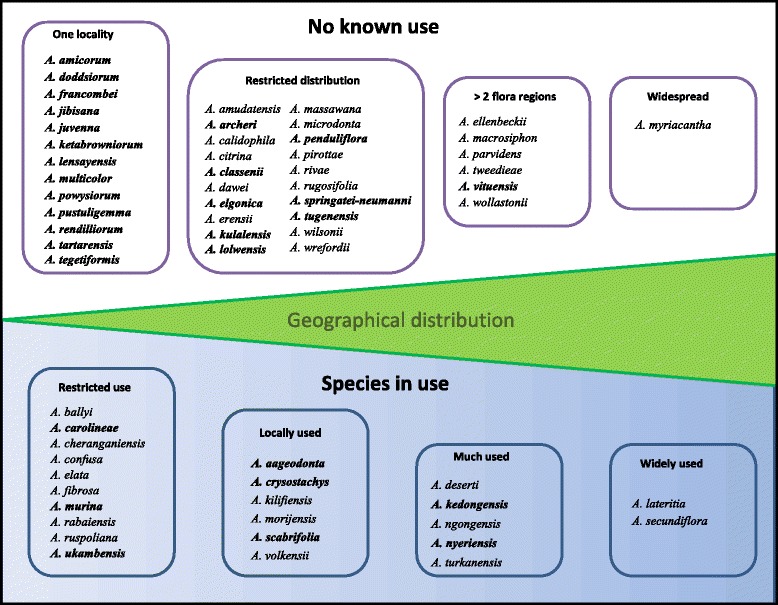
Extent of use and distribution of all Kenyan *Aloe* species; endemic species are indicated in bold. Based on own study, literature and herbarium records

Our data suggest that knowledge about the uses of *Aloe* species is commonly held among community members in Kenya, and knowledge is distributed evenly across ages and gender. That the Maasai and Kamba ethnic groups listed the highest numbers unique use records, might only be a reflection of the relative higher species diversity within their residential areas. Aloes are renowned internationally and some of the formally educated respondents claimed that the name of the local aloe (most often *A. secundiflora*) was *A. vera*, and that it was indigenous to Kenya. Public opinion about *A. vera* as “a wonder drug” has therefore been transferred to local aloes. In contrast to a general belief that all aloes are used wherever they grow [16] we could not find any documentation of use for 63 % of the Kenyan aloes (Fig. 5). The extent to which a species is uses is a result of the combination of availability and suitability for a particular purpose. Widespread species of *Aloe* are generally more frequently used than geographically restricted species, and uses are influenced by the growth form of the aloes. The shrubby *A. ngongensis*, *A. kedongensis* Reynolds and *A. nyeriensis* and the small tree *A. volkensii* Engl. are more suitable for fencing and hedging than the smaller and acaulescent species.

Species that are only found at one or few remote localities appear to be overlooked because people do not have easy access to the plants. However, some species that are relatively accessible are not used. Three (*A. classenii* Reynolds*, A. penduliflora* Baker and *A. vituensis* Baker) out of a total of 19 species documented in this study had no reported use. A few species had very limited use, such as *A. fibrosa* Lavranos & L.E.Newton and *A. elata*, for which the only reported use was as ornamentals. One special case is the very widespread *A. myriacantha* (Haw.) Schult. & Schult. f. (Fig. 5). Being a “grass aloe” it lacks the typical succulent leaves that people use. Succulent leaves have been positively correlated with the likelihood that an *Aloe* species is used for medicine [38]. Dying back in the dry season, people probably do not regard this as an aloe at all.

Local knowledge of natural resources has been recognised as an important element of effective conservation strategies in Kenya [37]. We found no significant connection between high numbers of recorded uses and perceived decreases in *Aloe* population sizes. Harvesting practices differ markedly in their impact on the *Aloe* plant: only small pieces of the leaves are used for the various medical uses while, in contrast, the whole plant is dug up when the stems are used for making beer, or the entire plant for fodder. However, we found no correlation between decrease in aloe populations and either the tradition of beer-making or the use aloes for fodder. This might indicate that local harvesting in general is done on a sustainable basis, and that habitat destruction due to increase in human and livestock populations poses a more urgent threat to *Aloe* species Kenya. Indeed, changes in land use were the most frequently given reasons for perceived declines in *Aloe* populations. Another factor that could be influential is that the most used species *Aloe secundiflora* is very common: a survey done by Mukonyi et al. (Mukonyi KW, Situma CA, Lusweti A, and Kyalo, S. Sustainable utilization of commercial *Aloe* resources in the drylands of Kenya 2005. Unpublished report) estimated the Kenyan population of commercial aloes at 129 million plants, 83 % of which were *A. secundiflora*. With such an abundant species, declining availability should be quickly noticeable. However, several respondents reported that they have to walk longer distances than before to collect their medicine from an *Aloe* population. As a consequence, aloes are more frequently planted in residential compounds to ensure the plants are accessible when they are needed (Fig. 4c). Local people are actively propagating seeds and replanting plantlets from other populations, conserving the local genetic diversity of *Aloe* species. Further, several informants responded that when moving from another area they had brought the aloes with them, indicating that the most-used aloes also are spread by humans. The succulent leaves allow aloes to survive for a long time out of soil, and the practice of moving aloes is well known [16]. *Aloe vera*, in particular, is an example with highly expanded distribution due to use, and was spread from the Arabian Peninsula along the trade routes to the Mediterranean, India, Americas and Caribbean [1, 38].

There is a close connection between the diversity of use and geographical distribution of *Aloe* species in Kenya (Figs. 3, 5). Not surprisingly, the wider the distribution range is for a species, the higher the number of uses documented for it. A species' range is also correlated with the number of ethnic groups that may use a species; in this study, *A. lateritia* and *A. secundiflora* were used by 8 and 7 ethnic groups, respectively (Fig. 2). At the other end of the scale, about 50 % of the species were used by only one ethnic group. In our study area, 16 of a total of 19 species were used (84 %), in contrast to the overall use in Kenya where only 23 out of 63 species of *Aloe* (37 %) are documented to be used. We propose that this disparity is due to the high number of rare species of *Aloe* in Kenya, which are often found in remote areas. In inhabited areas, the aloes undoubtedly play a very important role for the society, people and their livestock.

## Conclusions

Aloes are very important plants of use in Kenya. Their value is evident in the shared knowledge and use practices for species of *Aloe* throughout communities, regardless of age, gender, ethnic belonging, and place of residence. The ethnobotanical genus and species delimitation coincides with the scientific species concepts, while vernacular naming is closely linked to use and validates the use of vernacular nomenclature as a simple proxy for plant value. In Kenya, 37 % of *Aloe* species are used, of which the most important are the widespread *Aloe secundiflora* and *A. lateritia* and the most common uses, amongst all ethnic groups, are the treatment of malaria and poultry diseases. These findings agree remarkably with findings of studies in other regions of the *Aloe* distribution [4].

The use and exploitation of natural resources is often regarded as a threat to plant populations if the sustainability of harvesting practices has not been assessed. Illegal commercial harvesting is a grave threat to *Aloe* species in Kenya, whereas wild harvesting for local uses appears to be sustainable at current levels. The availability of useful *Aloe* species appears to be impacted more immediately by habitat loss, and species’ value has stimulated protection and propagation measures on a local scale.

**Table 2:** The unique uses of Aloes in the study area with reference to ethnic group. The uses are categorized into seven groups of use. Species are presented sequential from the most to the least used. K-Kamba, Ki-Kikuyu, Kii-Kisii, Ma-Maasai, Mb-Mbeere, Sa-Samburu, So-Somali, T- Taita, Tu- Tugen

Medicinal, humans	Ethnic group	Medicinal, livestock	Ethnic group	Fodder	Ethnic group	Fencing, soil conservation	Ethnic group	Local brew	Ethnic group	Cosmetic	Ethnic group	Other	Ethnic group
*A. aageodonta* L.E.Newton													
High blood pressure	K	chicken coughs	K									termite resistant	K
Syphilis	K											ornamental	K
Gonorrhoea	K											lethal	K
Malaria	K												
Diaphragm	K												
*A. ballyi* Reynolds													
												poisonous	Ta
												ornamental	Mb
*A. chrysostachys* Lavranos & L.E.Newton													
Malaria	Mb	Cattle wounds, fly repellent	Mb			Boundaries	Mb	Miti dawa	Mb				
Pneumonia	K	Chicken cough	K										
Diaphram	K												
*A. deserti* A. Berger													
Arthritis	Ma	chicken cough	K									Ornamental	Ma
Chest pain	K	chicken diarrhoea	K										
Gonorrhoea	Ma	chicken, bile treatment	T										
Malaria	K	goats, general treatment	K										
Pneumonia	Ma												
Retarded growth	Ma												
Tongue, inflammation	T												
*A. elata* S.Carter & L.E.Newton													
												ornamental	Ma
*A. fibrosa* Lavranos & L.E.Newton													
												ornamental	Ka
*A. kedongensis* Reynolds													
Typhoid	Ma			browsed by goats during drought	Ma	fences, hedging	Ma, Ka	Miti dawa	Ma			ornamental	Ma
Sexually transmitted diseases	Ma											decoration of sisal basket	Tu
Malaria	Ma, Ka, Ki												
Vomiting	Ma												
Diarrhoea	Ma												
*A. lateritia* Engl.													
Burns	Tu	Chicken diseases	K, T, Tu	Browsed by goats (/during drought)	Sa	Demarcating boundaries	Sa	Beer	Ka, Ma, Sa	Hair treatment	Sa	Not supposed to touch	Kii
Coughs	Ma	Chicken anti bodies	Kii	Browsed by sheep	Ma					Oil-glycerine	Sa	Attracts lightening	Kii
Diarrhoea	Tu	Goats diarrhoea	K							Skin lotion	Kii	Bed wetting	Tu
Eye infection	Sa	Sheep diarrhoea	K							pimples	Sa	Frost bite	Sa
Eye, allergy	Ki												
Eye, used when poison enters	Sa												
Malaria	T												
Pneumonia	Ma												
Ringworm	Ki, Kii, Sa												
Skin disorders	K, Kii, Tu												
Sleeping sickness	Tu												
Stomach ache	Ki												
Typhoid	Ma												
*A. morijensis* S.Carter & Brandham													
Pneumonia	Ma			Browsed by goats	Ma								
Ear alignment	Ma			browsed by sheep	Ma								
Medicinal, humans	Ethnic group	Medicinal, livestock	Ethnic group	Fodder	Ethnic group	Fencing, soil conservation	Ethnic group	Local brew	Ethnic group	Cosmetic	Ethnic group	Other	Ethnic group
*A. morijensis* (cont.)													
Skin itchy	Ma												
Vomiting	Ma												
Malaria	Ma												
*A. ngongensis* Christian													
Back ache	Ma			Browsed by goats	Ma	Boundaries	Ma	Beer	Ma				
Burns	Ma			Browsed by sheep	Ma	Fences, hedging	Ma						
Diarrhoea	Ma			Browsed by camels	Ma								
Ear alignment	Ma												
Gonorrhoea	Ma												
Itchy skin	Ma												
Malaria	Ma												
Pneumonia	Ma												
Skin disorders	K, Ma												
Stomach ache	Ma,												
Syphilis	Ma												
Ulcer	Ma												
Vomiting	Ma												
Wounds	Ma												
*A. nyeriensis* Christian in Verd.													
Back ache	Sa	Goats diseases, general	Sa	Browsed by camels	Sa	Fences, hedges	Sa						
Chest pain	Sa			Browsed by goats	Sa								
Clean blood	Sa												
Eye infection	Sa												
Gonorrhoea	Sa												
Release bladder	Sa												
Wounds	Sa												
Medicinal, humans	Ethnic group	Medicinal, livestock	Ethnic group	Fodder	Ethnic group	Fencing, soil conservation	Ethnic group	Local brew	Ethnic group	Cosmetic	Ethnic group	Other	Ethnic group
*A. scabrifolia* L.E.Newton & Lavranos													
												Gums for arrows	Ma
*A. secundiflora* Engl.													
Arthritis	Ma	Cattle disease, general	Sa	Goats browsing bark	Ma	Boundaries	Ma	Beer	Ma, Sa	Skin lotion	So	Goats die with eating	Sa
Backache	Ma	Cattle wounds, fly repellent	Mb	Eaten by livestock during drought	Sa, So	Demarcating roads	Ma	Miti dawa	T	Soap	T	Inflorescence eaten by children	Ma
Bile problems	Ma	Cattle, eye infection	Sa, So			Fences, hedging	Ma					Killing brown ticks	Tu
Burns	Ma, So	Chicken cough	K, Mb, T			Soil erosion	Ma					Ornamental	K, Ma, T
Cold, flue, coughs, Chest problems	K, Ma, Sa, So	Chicken white droppings/diarrhoea	K, So									Poison for arrows	K
Diabetes	So	Chicken, remove mite	K									Ritual	Ma
Diarrhoea, induce	Ma, T	Goats diarrhoea	K, Ma, Sa, So										
Diarrhoea, stopping of	Ma	Goat disease, general	K										
Eye infection, Trachoma conjunctivitis	Ma, Sa	Sheep's diarrhoea	K										
Gonorrhoea	Ma												
Headache	Ma												
High blood pressure	So												
Infections, cleaning of	Sa												
Itchy skin	So												
Kidney problems	Ma												
Liver problems	K												
Malaria	K, Ma, Mb, Sa, So, T, Tu												
Medicinal, humans	Ethnic group	Medicinal, livestock	Ethnic group	Fodder	Ethnic group	Fencing, soil conservation	Ethnic group	Local brew	Ethnic group	Cosmetic	Ethnic group	Other	Ethnic group
*A. secundiflora*cont.													
Oral thrush	Tu												
Pain relief	K												
Pancreas, swelling of	K												
Peptic ulcer	K												
Pneumonia	K, Mb, Sa, Tu												
Rachitis	K												
Ringworm	K, So, T												
Stiff muscles	K												
Stomach ache, Diaphragm problems	Ma, Sa, K, T												
Swelling of legs	K, Ma												
Tuberculosis	Ma												
Typhoid	Ma												
Ulcer, boils	K, So, Tu												
Vomiting	Ma, So,												
Wounds (cleaning fresh -)	Ma, Sa, T												
*A. volkensii* Engl.													
Pneumonia	Ma	east coast fever, goats	Ma	browsed by goats	Ma	boundaries	Ma	Beer	Ma			gums for arrows	Ma
		wounds, cattle	Ma			fences, hedging	Ma						
						wind breaking	Ma						
*A. ukambensis* Reynolds													
Diaphragm	K	chicken diseases	K										
Malaria	K												

## References

[1] Morton JF (1961). Folk uses and commercial exploitation of Aloe leaf pulp. Econ Bot.

[2] Crosswhite FS, Crosswhite CD (1984). Aloe vera, plant symbolism and the threshing floor: light, life and good in our heritage. Desert Plants.

[3] Atherton P (1998). First aid plant. Chem Brit.

[4] Grace OM, Simmonds MSJ, Smith GF, Van Wyk AE (2009). Documented utility and biocultural value of Aloe L. (Asphodelaceae): a review. Econ Bot.

[5] Rowley GD (1997). A History of Succulent Plants.

[6] Carter S, Lavranos JJ, Newton LE, Walker CC (2011). Aloes: The Definitive Guide. Royal Botanic Gardens.

[7] Grace OM (2011). Current perspectives on the economic botany of the genus Aloe L. (Xanthorrhoeaceae). S Afr J Bot.

[8] Carter S, Polhill RM (1994). Aloaceae. Flora of Tropical East Africa.

[9] Eggli U, Newton LE, Rowley GD (2001). CITES Aloe and Pachypodium checklist. Royal Botanic Gardens.

[10] Newton LE, Eggli U (2001). Aloe. Illustrated Handbook of Succulent Plants, Monocotyledons.

[11] Wabuyele E, Bjorå CS, Nordal I, Newton LE (2006). Distribution, diversity and conservation of the genus Aloe in Kenya. J East Afr Nat Hist Soc.

[12] McCoy T, Lavranos JJ (2007). Two significant new aloes from Kenya. Cact World.

[13] Newton LE (2011). Two new species of Aloe in Kenya. Bradleya.

[14] Wabuyele E, Kyalo S (2008). Sustainable Use of East African Aloes: The case of Commercial Aloes in Kenya.

[15] Tambo OHA (1991). A phytochemical investigation of three species of Aloe selected for possible commercial exploitation. M.Sc thesis.

[16] Oldfield S. Review of Significant Trade East African Aloes. CITES; 2003. PC14 Do. 9.2.2 Annex 4. https://cites.org/sites/default/files/eng/com/pc/14/E-PC14-09-02-02-A4.pdf

[17] Lubia IK, Kyalo SN, Mukonyi KW, Lusweti AM, Situma CA. Strategy for conservation and management of commercial Aloe species in Kenya. Kenya Wildlife Report. 2008. www.kws.go.ke/download/file/fid/1405. Accessed 1 Sept 2015

[18] Nyamora P (1986). Medicinal plant given presidential protection. Daily Nation (Kenya).

[19] Newton LE (1994). Exploitation and conservation of Aloes in Kenya.

[20] Anonymous. Wildlife (Conservation and management) (Aloe species) Regulations. 2007 [L.N. 403/2007]. http://faolex.fao.org/docs/pdf/ken101511.pdf. Accessed 1 Sept 2015

[21] Mukoyon KW, Owuor B, Chikamai BN, Wabuyele E (2001). A review and appraisal of the Aloe recourses in Kenya; utilization and development status.

[22] Newton LE, Russo L (2004). Succulent Plant Utilisation and Conservation in Eastern Tropical Africa. Proceedings of The Succulent Plants of Eastern Africa: History, Botanical Exploration and Research.

[23] Newton LE (2007). Goodbye to the type locality of Aloe ballyi. A sad tale of habitat destruction in Kenya. CactusWorld.

[24] The IUCN red list of treatened species. http://www.iucnredlist.org/search. Accessed Jan 2015

[25] Holman EW (2002). The relation between folk and scientific classification of plants and animals. J Classif.

[26] Grace OM, Simmonds MSJ, Smith GF, Van Wyk AE (2008). Therapeutic uses of Aloe L. (Asphodelaceae) in Southern Africa. J Ethnopharmacol.

[27] Neuwinger HD (1994). Afrikanische Arzneipflanzen und Jagdgifte Chemie, Pharmakologie, Toxikologie.

[28] Oketch-Rabah HA, Dossaji SF, Mberu EK (1999). Antimalarial activity of some Kenyan medicinal plants. Pharm Biol.

[29] Waihenya RK, Mtambo MMA, Nkwenguila G, Minga UM (2002). Efficacy of crude extract of Aloe secundiflora against Salmonella gallinarum in experimentally infected free-range chickens in Tanzania. J Ethnopharmacol.

[30] Hindmarsh L (1982). A notebook for Kenyan Dyers.

[31] Kokwaro JO (1993). Medicinal plants of East Africa.

[32] Maundu PM, Ngugi GW, Kabuye CHS (1999). Traditional Food Plants of Kenya.

[33] Anonymous. R version 3.1.2 for Windows. The R foundation for statistical computing; 2014. http://cran.r-project.org.

[34] Morgan WTW (1981). Ethnobotany of the Turkana: use of plants by a pastoral people and their livestock in Kenya. Econ Bot.

[35] Bussmann RW (2006). Ethnobotany of the Samburu of Mt. Nyiru, South Turkana, Kenya. J Ethnobiol Ethnomed.

[36] Grade JT, Tabuti JRS, Van Damme P (2009). Ethnoveterinary knowledge in pastorial Karamoja Uganda. J Ethnopharmacol.

[37] Jeruto P, Lukhoba C, Ouma G, Otieno D, Mutai C (2007). An ethnobotanical study of medicinal plants used by the Nandi people in Kenya. J Ethnopharmacol.

[38] Grace OM, Buerki S, Symonds MRE, Forest F, van Wyk AE, Smith GF, Klopper RR, Bjorå CS, Neale S, Demissew S, Simmonds MSJ, Rønsted N (2015). Evolutionary history and leaf succulence as explanations for medicinal use in aloes and the global popularity of Aloe vera. BMC Evol Biol.

